# Simulations for Mechanical Ventilation in Children: Review and Future Prospects

**DOI:** 10.1155/2013/943281

**Published:** 2013-03-07

**Authors:** Olivier Flechelles, Annie Ho, Patrice Hernert, Guillaume Emeriaud, Nesrine Zaglam, Farida Cheriet, Philippe A. Jouvet

**Affiliations:** ^1^Pediatric ICU, Sainte-Justine Hospital, University of Montreal, Montreal, QC, Canada H3T 1C5; ^2^Pediatric and Neonatal ICU, MFME Hospital, Fort de France, 97261 Martinique, France; ^3^Research Center of Sainte-Justine Hospital, Montreal, QC, Canada H3T 1C5; ^4^École Polytechnique de Montréal, Montreal QC, Canada H3T 1J4; ^5^Soins Intensifs Pédiatriques, Hôpital Sainte Justine, 3175 Chemin Côte Sainte Catherine, Montréal, QC, Canada H3T 1C5

## Abstract

Mechanical ventilation is a very effective therapy, but with many complications. Simulators are used in many fields, including medicine, to enhance safety issues. In the intensive care unit, they are used for teaching cardiorespiratory physiology and ventilation, for testing ventilator performance, for forecasting the effect of ventilatory support, and to determine optimal ventilatory management. They are also used in research and development of clinical decision support systems (CDSSs) and explicit computerized protocols in closed loop. For all those reasons, cardiorespiratory simulators are one of the tools that help to decrease mechanical ventilation duration and complications. This paper describes the different types of simulators described in the literature for physiologic simulation and modeling of the respiratory system, including a new simulator (SimulResp), and proposes a validation process for these simulators.

## 1. Introduction

Mechanical ventilation is a lifesaving therapy which is associated with complications such as baro-, volo-, and biotrauma, ventilation-induced pneumonia and laryngeal stenosis [1–3]. These complications can be decreased through the use of a protective ventilation strategy for acute lung injury [4] and the use of protocols to reduce the duration of weaning [5]. These protocols include a set of patient-specific therapy instructions that change according to the patient's cardiorespiratory condition. Because it is difficult to test each modification/improvement that is made to the protocol on real patients, there is an increasing interest in the development of physiologic models that simulate cardiorespiratory responses to modifications of mechanical ventilation settings.

We reviewed the literature and report our experience on physiologic simulation and modeling of the respiratory system.

## 2. Simulators of Mechanical Ventilation in the Literature

Simulation is a strategy to replace or amplify real experiences with another experience that evokes certain aspects of the real world in constant interaction with the user [6]. Three categories of simulation have been described: simulation of patient signs and symptoms, anatomic simulation of the lung, and physiologic simulation. For each of these categories, there are several types of simulators with different goals, each of them with their advantages and disadvantages. Currently, the different types of simulators are as follows: (1) simulators to assess ventilator performance; (2) simulators used to teach physiology and ventilation management; (3) simulators for ventilation management recommendations; (4) simulators for the development of clinical decision support systems (Table 1) [7]. The different types of simulators reported in the literature are further described below.

### 2.1. Simulators for the Testing of Ventilator Performance

To assess ventilator performances, simulators of simple lung mechanics are used to simulate the passive respiratory system. These simulators can be homemade [8, 9] or more sophisticated. The latter are produced commercially (e.g., the 5601i Adult/Infant PneuView (*Michigan Instruments Inc*., Grand Rapids, US), IngMar ASL5000 lung simulator (*IngMar Medical, Ltd*., Pittsburgh, US), Series1101 Breathing Simulator (*Hans Rudolph, Inc.,* Shawnee, US)). Lung compliance, airways resistance, lung volume, and respiratory rates are set by investigators according to the age and disease being simulated [10–12]. These test lungs do not reflect the complexity of lung mechanics (variation of lung and chest wall compliance according to lung volume, variation of resistance with lung volume and airway flow, intrinsic PEEP), do not include blood gas exchange analysis, and are of limited value in simulating the cardiorespiratory effects of a modification of mechanical ventilation settings. 

One simulator is dedicated to facilitating the choice of a ventilator by stakeholders according to specific criteria. These criteria are entered, and the software selects the best ventilator (Purchasing Decision Tool available on the website http://www.ventworld.com/).

### 2.2. Simulators for Education Purposes

At present, training on mechanical ventilation is mainly delivered at the bedside. This training is generally unsatisfying for junior trainees who are frequently the first-line prescribers [13]. However, in contrast with clinical practice at the bedside, making a wrong decision on a simulator promotes clinician learning without harming the patient. Corbridge et al. demonstrated in a randomized clinical trial that students prefer using simulation to online learning [14]. In veterinary medicine, Keegan et al. created a “virtual ventilator” and tested it on 109 veterinary students. They demonstrated that learning with a simulator is as effective as with animals and that the students preferred starting with a simulator before managing animals [15]. Recognizing that interactive learning opportunities are more effective than didactic ones it can be concluded that simulators are helpful in teaching about mechanical ventilation. 

#### 2.2.1. Respiratory Mechanics Simulators

This kind of simulator is used to assess ventilators performance and can also be used to teach students the basics of respiratory system mechanics and the technical characteristics of ventilators and modes of ventilation [16]. 

#### 2.2.2. Ventilator Simulators

These are computer simulations of a ventilator available online, the aim of which is to present and teach principles of mechanical ventilation. Students can choose and change patient lung features and then see the effect of different ventilator settings. Those simulators can be homemade.

-  “Mechanical Ventilation Simulator” (http://www.ohsu.edu/academic/picu/medialab/vent/). This uses very simple software, and its purpose is to explain the effect of ventilator settings on arterial blood gases for only five different patients (normal 10 kg child to 100 kg adult with acute respiratory distress syndrome (ARDS)).
-  The “virtual ventilator” developed by Takeuchi et al. is a more complex program to teach ventilation to physicians [17]. The “Virtual Ventilator” is able to reproduce many characteristics of a patient and of different ventilation modes.
-  Evita_trainer: this simulator has been developed by Draeger Medical (Lübeck, Germany) to teach how to use their company's ventilators. The screen mimicks [*sic*] the Evita IV interface and caregivers can modify ventilator settings and have access to the resulting ventilation curves (pressure-time, flow-time,…).

#### 2.2.3. Physiologic Cardiorespiratory Simulators

This type of simulator is able to reproduce cardiorespiratory physiology and provide arterial blood gas values. 

**Figure 1:**
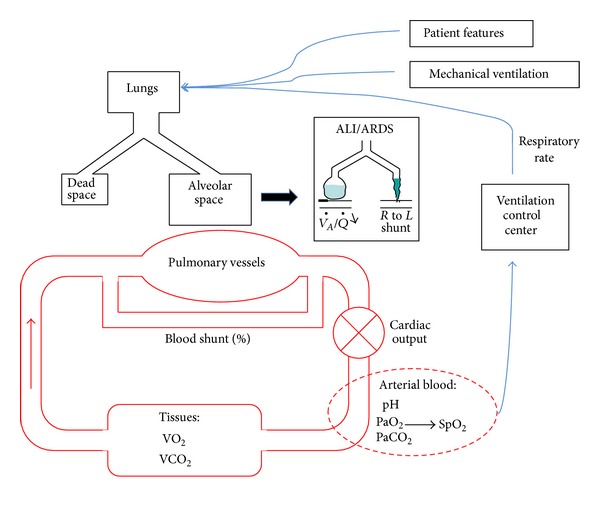
Schematic presentation of the cardiorespiratory model developed by Dickinson and used in the SimulResp simulator. ALI: acute lung injury, ARDS: Acute Respiratory Distress Syndrome.

Most of simulators are based on a three-compartment model of respiration: the capillary compartment as the “ideal” compartment where gas exchange takes place, the right-to-left shunt, and dead space. First validated on healthy patients, those simulators are also able to simulate various cardiopulmonary diseases, and some of them also simulate the impact of positive pressure ventilation. If positive pressure ventilation is accurately simulated, these simulators can be used to predict the impact of a modification of ventilation setting on blood gases (ventilatory effect forecast). These simulators include the following:

- The MacPuf simulator developed by Dickinson. The Dickinson model takes into account blood circulation, the gas exchange system, ventilation control, and tissue metabolism. The cardiorespiratory condition of a patient is simulated through the setting of 26 parameters that can be set by users within physiological ranges observed in intensive care. In this model, blood flow is simulated by several steps with arterial, tissue, and venous passage (Figure 1). Gas exchange between blood and tissues is specified taking into account blood dissociation curves for oxygen and carbon dioxide to simulate the transfers of oxygen and CO_2_. The pulmonary circulation is modeled in three different areas with the capillary compartment as the “ideal” compartment where gas exchange takes place. Another compartment simulates a right-to-left shunt. The third compartment is dead space which has no contact with blood. Gas exchange and respiratory mechanics are simulated according to the alveolar ventilation and gas-exchange time but also in terms of respiratory rate, compliance, lung capacity, and oxygen saturation. It is also possible to define a left-to-right shunt if needed. In spontaneous ventilation, ventilation and respiratory rate are regulated by hypercapnia and hypoxia through simulation of chemoreceptors and also by acid-base status. This model is able to predict lactic acid production in hypoxia states. The model is capable of mimicking artificial ventilation with setting of FiO_2_ and positive end-expiratory pressure (PEEP) or mean airway pressure (Paw) which impacts upon venous return to the lungs and cardiac output [18]. This model has been used as the matrix for several other simulators.
- HUMAN simulator simulates cardiovascular, renal, temperature regulation and some hormonal functions [19].
- VentSim includes a ventilator component (volume-cycled, constant-flow ventilator), an airway component, and a circulation component. This simulator includes arterial and venous blood gases [20]; validation of VentSim on simulated patients shows a good match between the blood gas provided by the simulator and the clinical range. However, a comparative assessment with data from actual ventilated patients is missing, and the ability to simulate unstable patients (as frequently encountered in intensive care units) is questionable.
- SOPAVent: Wang et al. [21] developed a simulator based on a 3-compartment physiological model. The model works presently with stable patients. Other simulators use a multicompartment model that needs to set the ventilation-perfusion ratio for each compartment.
- SimuVent [22] considers the interaction between simulator and ventilator. The interface is graphical, so the software is easy to use.
- VO2.htm needs to set the ventilation-perfusion ratio for each compartment. There is no validation for this model. The software is available online (http://www.siumed.edu/medicine/pulmonary/VO2.htm) [23].
- Nottingham Physiology Simulator (NPS). The model from Das et al. [24] is built to be a “ventilatory effect forecast,” NPS with Matlab [25], based on NPS. The lungs are modelled as a dynamical system that includes external equipment (e.g., a mechanical ventilator), anatomical and alveolar dead spaces, and ventilated, perfused alveoli. To model the heterogeneity of patient populations and diseases, the authors integrated uncertainty and variability into the equations and a mechanism to ensure that the model prediction is within physiological range. However, currently, these ranges do not include all conditions observed in clinical practice (pH between 7.3 and 7.5; PO2 between 9 and 15 KPa).
- ARDS simulator. Reynolds et al. [26] developed a mathematical model of pulmonary gas exchange under inflammatory stress. This approach needs to set 63 different parameters. The first tests of the simulator are encouraging.

#### 2.2.4. High-Fidelity Patient Simulators

 These simulators are physical models close to real life, thereby facilitating learning through the reproduction of reality in three dimensions. This type of simulator can be connected to a mechanical ventilator to teach basic respiratory physiology, but their physical characteristics do not simulate lung mechanics, and they are best used for an overall patient assessment [27]. They are used to train teams in various emergency situations and have been shown to reduce errors and improve outcomes in neonatal care [28]. Commercialized high-fidelity patient simulators include SimMan by *Laerdal Medical*, Stavanger, Norway, and human patient simulator by *CAE Healthcare*, Montreal, Canada. 

### 2.3. Simulators for Ventilation Management Recommendations

Intelligent Ventilator: Rees et al. developed a model that includes oxygen and carbon dioxide gas exchange and storage modelling and a linear model of lung mechanics. This model is combined with penalty functions describing clinical preference toward the goals and side effects of mechanical ventilation in a decision theory approach. The model is fitted to patient's clinical conditions via several measurements including arterial blood sample drawn at the clinical FiO_2_; assessment of O_2_ consumption and CO_2_ production; measurement of anatomical dead space from volumetric capnography; measurement of pulmonary shunt from a procedure of varying FiO_2_ in steps and measuring ventilation, metabolism, and oxygenation status at each step; calculation of dynamic compliance from PIP, PEEP, and Vt. After the model is fitted to the patient, the clinical decision support system connected to the model tests several combinations of FiO_2_, respiratory rate, and tidal volume and proposes the settings with the best clinical impact. The simulations obtained were shown to be close to ARDS network recommendations in a retrospective study using data from real patients [29, 30].

### 2.4. Simulators for the Development of Computer-Driven Protocols in Mechanical Ventilation (Clinical Decision Support Systems and Closed-Loop Explicit Computerized Protocols)

In aviation, a domain with similar safety issues to medicine, flight simulators are used in the development of computer-driven protocols used by autopilot. In medicine, models and simulations are nowadays integrated in research protocols, and some results obtained from simulation are taken into account with those obtained from basic science research and clinical trials [31, 32].

The mismatch between human ability and the vast amount of data and information in intensive care at the bedside contributes to the variation in clinical practice, as decisions are made applying different data constructs and different knowledge/expertise. To help clinicians in their decision making, to standardize but also personalize care, computer-driven protocols have been developed for the management of mechanical ventilation [33]. A computer-driven protocol can work in a closed-loop and/or open-loop mode. In the former (closed loop), the computer implements its recommendation without caregiver intervention, through so-called closed-loop explicit computerized protocols (CL-ECPs); in the latter (open loop), the computer provides recommendations that can be approved or not by caregivers; these are called clinical decision support systems (CDSS). Systematic testing and validation of CDSS and CL-ECPs is a critical phase that needs a specific simulator and testing plan [34]. The simulator should have realistic physiological behaviours. Several research teams and companies are working on such platforms, although none are currently commercialized for this purpose [33].

## 3. Development of a Cardiorespiratory Simulator for Computer-Driven Protocols in Mechanical Ventilation: SimulResp

To complete a platform dedicated to the development of a Computer-Driven protocol for mechanical ventilation in children, we developed a cardiorespiratory simulator. This simulator was created to test and validate a Computer-Driven protocol for the management of ARDS and to train caregivers when this protocol will be in use [33]. 

### 3.1. Mathematical Model Used

**Figure 2:**
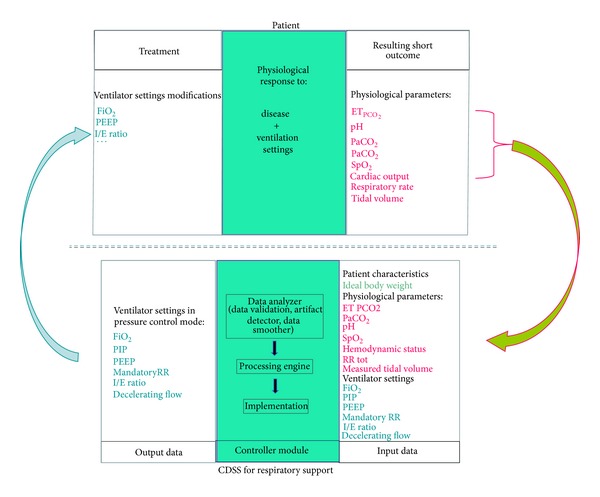
Schematic presentation of the interaction between a simulator and a clinical decision support system during the R&D phase. PaCO_2_: partial pressure of carbon dioxide, ET PCO_2_: end-tidal PCO_2_, PaO_2_: partial pressure of oxygen, SpO_2_: saturation of peripheral oxygen, FiO_2_: fraction of inspired oxygen, PIP: positive inspiratory pressure, PEEP: positive end-expiratory pressure, I/E ratio: inspiratory/expiratory ratio, RR: respiratory rate, CDSS: clinical decision support system.

The platform for the computer-driven protocol for mechanical ventilation includes software that collects electronically compiled clinical data from a patient (from monitors, ventilator, IV pumps, etc.) and transforms these data into a recommendation for mechanical ventilation setting(s), either displayed on a screen (CDSS) or modified directly without caregiver intervention (CL-ECPs). To develop and validate the computer-driven prescriptions, a simulator is needed. The simulator consists of a mathematical model of cardiorespiratory physiology coded into a software program that feeds the Computer-Driven protocol platform (Figure 2). According to the specifications of the computer-driven protocol [33], the mathematical model needs the following characteristics: (1) to simulate spontaneous and artificial ventilation, (2) to be able to simulate adult and child parameters including various ages and weights, (3) to be able to simulate various conditions including lung diseases (variation of compliance, resistance, dead space, residual functional capacity), hemodynamic instability, and (4) to deliver the following cardiorespiratory and general parameters: arterial blood gas values (PaO_2_, pH, PaCO_2_, SpO_2_), end tidal PCO_2_ (ET_PCO2_), cardiac output, heart rate, respiratory rate, blood left-to-right shunt, blood right-to-left shunt, tissue oxygen consumption, tissue CO_2_ production, body temperature (Figure 2). All these parameters were provided by the three-compartment model developed by Dickinson (see above) [18], and the source code of the simulator was in the public domain. This model is relatively simple, but offers a good representation of reality [35].

### 3.2. Technical Aspects of SimulResp

**Figure 3:**
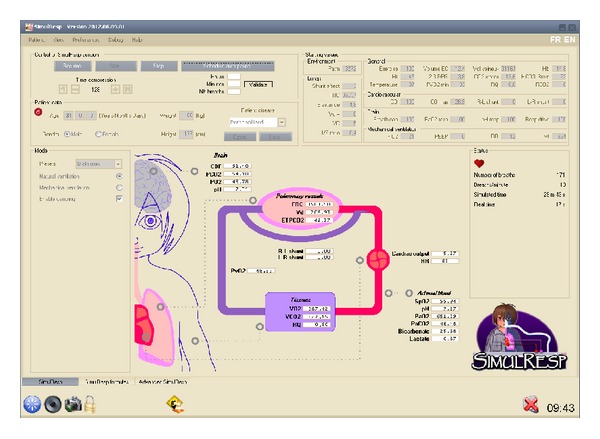
Picture of the cardiorespiratory simulator, SimulResp.

The initial computer language was in FORTRAN. Simulator source code was translated into C++, a computer language created for use over a long period of time (estimated use 20 years). This translation was compiled as a “dynamic link library” (DLL) that allows different programs to share codes and provides resources necessary to perform various tasks in harmony. A visual interface was developed (Figure 3) to facilitate user interaction with the simulator. A time-compression system was included in order to simulate 24 hours in a short period of time (2 min) if necessary. This characteristic was developed in order to test computer-driven prescription that may only be triggered a few times a day. This can be the case of a PEEP protocol for example. Several categories of patients with predefined criteria were created: normal, ARDS, chronic obstructive pulmonary disease, asthma, hemodynamically unstable, and custom.

### 3.3. SimulResp Validation Procedure

For any simulator, it is essential to incorporate into the design process a validation procedure. This is the step which ensures that the simulator meets its goals and that the results obtained are in a range of acceptable accuracy for the area studied. The credibility of a model depends on the quality of the validation. Validation should give clear evidence of its applicability and reliability [25]. The validation of SimulResp has already started [36] and includes the following steps.

 Tests in Spontaneous Ventilation. Accuracy is first tested in spontaneous ventilation with the simulation of healthy subjects. Accuracy is assessed using the correlation coefficient between blood gases obtained by the simulation and physiologic values published for the patient age, weight, height, and gender [37]. After this test, robustness is tested by comparing specific cardiorespiratory conditions described in the scientific literature to results obtained with the simulator. One example of a clinical condition that is tested is an increase in atmospheric pressure from 1 to 4.7 ATA [38]. Blood gases obtained from these studies are compared to the values obtained from SimulResp using the patient characteristics described in the scientific literature. The correlation between expected and observed values is studied using “*r*” Pearson correlation coefficient.

 Clinical Validation. Data from mechanically ventilated patients are compared to SimulResp prediction. A comparison is made between expected and observed steady-state arterial blood gases in response to changes in ventilator settings [21]. This patient data is obtained either from a retrospective electronic clinical database or from a prospective clinical study. In our research program, we plan to clinically validate SimulResp using both methodologies.

### 3.4. SimulResp Limitations

Currently, the software does not simulate children under 7 years of age, which is problematic as 2/3 of the children admitted to the pediatric intensive care unit and mechanically ventilated are less than 2 years old [39]. A new version of the software including the mathematical model for children of 1 month to 8 years is in development.

In the mathematical model chosen, there are approximations, which were chosen empirically by trial and error, to avoid damping phenomena. We will probably have to adjust the mathematical model during the validation phase. These modifications of the mathematical model in response to unsuccessful validation tests highlight the continuous improvement process that is necessary when the simulation of complex systems is attempted. This process ends when the simulator is mature, that is, close approximating clinical behavior.

## 4. Conclusion and Future

Over the last 30 years, simulators have been used in intensive care units for teaching respiratory physiology and testing ventilator performance. Recently, technical advances, especially in computer science, have increased the calculation power of computerized systems. This has contributed to the development of a new generation of simulators. SimulResp is a new simulator based on a 3-compartment lung model embedded in a Computer-Driven protocol. In clinical practice, this kind of simulator can be helpful for training on mechanical ventilation, in the prediction of patient outcome based on clinical status and settings of respiratory support (ventilatory effect forecast), and in combination with a clinical decision support system, it can help physicians to set ventilator parameters. The validation procedure is a major issue because the credibility of a model depends on the quality of its validation. Validation is part of the simulator refinement process and needs dedicated clinical databases and prospective clinical studies. With such an approach, simulators will help in the development of ventilation management protocols as well as in training caregivers to use these protocols in order to reduce adverse effects and costs due to prolonged mechanical ventilation.

## Figures and Tables

**Table 1:** Different types of simulators available in 2012.

Different simulators	Aim							
	Teaching		Ventilator performance	Ventilatory effect forecast	Ventilation management advisor	Research and development	Adult	Child
	Physiology	Ventilation						
Mechanics simulators								
Homemade [8–11]		*√*	*√*				*√*	*√*
PneuView*		*√*	*√*				*√*	*√*
ASL5000**		*√*	*√*				*√*	*√*
Series 1101***		*√*	*√*				*√*	NA
Ventilator simulators								
Virtual ventilator [17]		*√*					*√*	NA
Purchasing decision tool			*√*				*√*	NA
Evita_trainer^†^		*√*					*√*	*√*
Mechanical ventilation simulator		*√*					*√*	*√*
Physiology cardiorespiratory simulators								
MacPuf [18]	*√*						*√*	*√*
HUMAN [19]	*√*						*√*	NA
VentSim [20]				*√*	*√*		*√*	NA
SimuVent [22]	*√*	*√*				*√*	*√*	NA
Nottingham physiology simulator (NPS) [24]				*√*			*√*	NA
Intelligent ventilator [29]				*√*	*√*		*√*	NA
VO2.htm [23]	*√*						*√*	NA
SOPAVent [21]				*√*	*√*		*√*	NA
NPS + Matlab [25]				*√*			*√*	NA
ARDS simulator [26]	*√*	NA					*√*	NA
SimulResp [33]	*√*	*√*		*√*	*√*	*√*	*√*	*√*
High-fidelity patient simulators								
SimMan^‡^		*√*					*√*	*√*
Human patient simulator^‡‡^		*√*					*√*	*√*

Nonexhaustive list. NA: information not available. *Michigan Instruments Inc., Grand Rapids, US. **IngMar Medical, Ltd., Pittsburgh, US. ***Hans Rudolph, Inc., Shawnee, US. ^†^Draeger, Siegen, Germany. ^‡^Laerdal Medical, Stavanger, Norway. ^‡‡^CAE Healthcare, Montreal, Canada.
